# Allelic Variation Analysis at the Vernalization Response and Photoperiod Genes in Russian Wheat Varieties Identified Two Novel Alleles of *Vrn-B3*

**DOI:** 10.3390/biom11121897

**Published:** 2021-12-17

**Authors:** Alina Berezhnaya, Antonina Kiseleva, Irina Leonova, Elena Salina

**Affiliations:** 1 The Federal State Budgetary Institution of Science Federal Research Center Institute of Cytology and Genetics, Siberian Branch of the Russian Academy of Sciences (ICG SB RAS), Prospekt Lavrentyeva 10, 630090 Novosibirsk, Russia; antkiseleva@bionet.nsc.ru (A.K.); leonova@bionet.nsc.ru (I.L.); salina@bionet.nsc.ru (E.S.); 2 Kurchatov Genomics Center, Institute of Cytology and Genetics SB RAS, Prospekt Lavrentyeva 10, 630090 Novosibirsk, Russia

**Keywords:** common wheat, Vrn and Ppd genes, heading time, flowering locus T

## Abstract

Heading time is an important agronomic trait affecting the adaptability and productivity of common wheat. In this study, 95 common wheat varieties from Russia and the late-maturing breeding line ‘Velut’ were tested for allelic diversity of genes having the strongest effect on heading. In this research, allelic variation at the *Ppd-D1, Vrn-A1, Vrn-B1, Vrn-D1,* and *Vrn-B3* loci was tested. The *Vrn-B1* and *Vrn-B3* loci provided the largest contribution to genetic diversity. We found two novel allelic variants of the *Vrn-B3* gene in the studied varieties. Ten varieties carried a 160 bp insertion in the promoter region, and the breeding line ‘Velut’ carried a 1617 bp insertion. These alleles were designated *Vrn-B3e* and *Vrn-B3d*, respectively. The analysis of the sequences showed the recent insertion of a retrotransposon homologous to the LTR retrotransposon (RLX_Hvul_Dacia_ RND-1) in the *Vrn-B3d* allele. Plants with the *Vrn-B3e* and the ‘Velut’ line with the *Vrn-B3d* allele headed later than the plants with the wild-type allele; among these plants, ‘Velut’ is the latest maturing wheat variety. Analysis of the gene expression of two groups of lines differing by the *Vrn-B3* alleles (*Vrn-B3d* or *vrn-B3*) from the F_2_ population with ‘Velut’ as a parental line did not reveal a significant difference in the expression level between the groups. Additional research is required to study the reasons for the late maturation of the ‘Velut’ line. However, the studied wheat varieties could be used as a potential source of natural variation in genes controlling heading times.

## 1. Introduction

The yield potential of wheat largely depends on the duration of each of the developmental phases. The heading time of wheat is mainly determined by the allelic composition of vernalization (*Vrn*) and photoperiod response (*Ppd-1*) genes [1]. The *Vrn-B3* gene (also known as *TaFT1*) is the central integrator of the vernalization and photoperiod pathways [2]. Under the control of the *Vrn-1*, *Vrn-2*, and *Ppd-1* genes, *Vrn-B3* is expressed in leaves and then moves to the shoot apical meristem and promotes flowering [2,3,4].

The *Vrn-1* genes controlling the wheat vernalization requirement were mapped to homologous chromosome group 5 [5]. The presence of at least one dominant *Vrn-1* allele in hexaploid wheat results in spring growth habit. The dominant *Vrn-A1* is epistatic to *Vrn-B1* and *Vrn-D1,* which are associated with a slight vernalization response [6]. Genetic variation at the *Vrn-1* loci is one of the most important factors affecting heading time in wheat. Plants with the dominant *Vrn-A1* allele flower earlier than those with dominant *Vrn-D1* or *Vrn-B1* [7]. The dominant alleles at the *Vrn-A1* locus are characterized by molecular variations (insertions and/or deletions) within the promoter region or by large intron deletions [8,9]. The dominant *Vrn-B1* and *Vrn-D1* alleles are also associated with deletions within the first intron [9]. Studies of the *Vrn-1* composition of Russian spring wheat showed that the combination of the vernalization-insensitive allele *Vrn-A1a* with the dominant *Vrn-B1a* or *Vrn-B1c* allele was the most frequent [10]. These two dominant alleles were found to be equally frequent: 47.6% of the examined varieties had the *Vrn-B1a* allele, and 40.4% had the *Vrn-B1c* allele. In contrast, in the analysis of 245 European spring wheat varieties, the *Vrn-B1c* allele was detected only in 16 varieties, mainly from Eastern European countries (Shcherban et al. 2015).

The sensitivity of wheat to photoperiod is mainly affected by *Ppd-1* genes, localized on 2A (*Ppd-A1*), 2B (*Ppd-B1*), and 2D chromosomes (*Ppd-D1*) [11]. Plants with the dominant *Ppd-1* are insensitive to the day length, which results in early heading under conditions of both short and long days. Worland (1998) reported that the *Ppd-D1* gene has the strongest effect. The dominant *Ppd-D1a* allele with a 2 kb deletion upstream of the coding region is associated with photoperiod insensitivity. Photoperiod-insensitive alleles predominate at southern latitudes, while wheat varieties grown in northern regions are usually sensitive to the photoperiod [12,13]. The presence of photoperiod-sensitive alleles significantly delays heading, especially under short-day conditions [14].

The *Vrn-B3* gene *(TaFT1*) is a key gene acting as an integrator of several autonomous pathways. The common wheat *Vrn-B3* gene mapped on chromosome 7B is homologous to the Arabidopsis *FT1* gene [2]. *FT*-like genes belong to the PEBP gene family and control flowering time in dicot and monocot plants [15]. There are four allelic variations that have been characterized, including the recessive *vrn-B3* allele. The dominant *Vrn-B3a* allele contains a 5295 bp retrotransposon insertion 591 bp upstream of the coding sequence [2]. The *Vrn-B3c* allele differs from *Vrn-B3a* by two small deletions (4 bp and 20 bp) inside the retrotransposon region [16]. Both alleles have similar levels of expression. The other dominant allele, designated *Vrn-B3b*, contains an insertion of 890 bp and is characterized by a lower expression level compared with the wild-type allele, associated with a delay in heading. At present, the natural variation of the *Vrn-B3* gene among Russian common wheat has not been sufficiently investigated, as most of the studies have focused on allelic variation at the *Vrn-1* and *Ppd-1* genes.

In this research, 95 spring common wheat varieties adapted to Siberian environments were tested for their allelic composition at the *Ppd-D1*, *Vrn-A1*, *Vrn-B1*, *Vrn-D1*, and *Vrn-B3* loci, which presumably are the main genetic factors involved in heading time development.

## 2. Materials and Methods

### 2.1. Plant Materials

Ninety-five Russian common wheat varieties adapted to the Ural and Siberian region growing conditions were used (Table S1). Phenotyping was conducted in 2016 and 2017 at the experimental field of the Federal Research Centre Institute of Cytology and Genetics SB RAS in Novosibirsk. The late-ripening breeding line ‘Velut’ was also included in the analysis. The origin of the ‘Velut’ line is shown in the Figure S1 [17,18,19,20]. The F_2_ population used in this study was developed from a cross between wheat varieties with different *Vrn-B3* alleles (‘Velut’ and Tobolskaya).

Varieties were sown in accordance with the systematic method of sample location in two replicates on plots of 1 m^2^, 60 grains per replicate, and between-row spacing 25 cm. The soil in the field was leached chernozem. Heading time was measured as the number of days from seedling emergence to the date when 1/2 of spike emerged from the flag leaf.

### 2.2. PCR Amplification

Genomic DNA was extracted from 3 to 4 seedlings following a modified protocol described by Plaschke et al. [21].

Allelic variation at the *Ppd-D1*, *Vrn-A1*, *Vrn-B1, Vrn-B3*, and *Vrn-D1* loci was determined using previously reported specific primers (Table 1). PCR amplification was performed as described in these papers. The 20 µL PCR mixture contained 2X HS-Taq PCR-Color (Biolabmix, Novosibirsk, Russia), 0.25 μmoles each of the reverse and forward primers, DNA at a concentration of 5 ng/μL, and sterile water.

The PCR amplification conditions with the primers designed in this study (Table 1) were as follows:

1. FTpr-F//R: 94 °C for 4 min to denature the DNA, 35 cycles at 94 °C for 10–40 s, 61 °C for 30 s, and 68 °C for 2.5 min, followed by a final extension at 72 °C for 10 min. The reaction was performed with 2X LR HS-PCR-Color (Biolabmix, Novosibirsk, Russia);
2. VRN-B3d-F//R: 95 °C for 5 min; 35 cycles of 95 °C for 45 s, 62 °C for 1 min, 72 °C for 1 min, and a final extension at 72 °C for 10 min;
3. VRN-B3e-F//R: 95 °C for 5 min, 35 cycles of 95 °C for 15 s, 59 °C for 30 s, 72 °C for 30 s, and a final extension at 72 °C for 5 min.

### 2.3. DNA Sequencing and Analysis

PCR products were isolated from the 2% agarose gel using a Zymoclean™ Gel DNA Recovery Kit (ZymoResearch, Irvine, CA, USA) and directly sequenced using a BigDye Terminator v.3.1 kit (Applied Biosystems, Forster City, CA, USA). Sequencing products were analyzed using an ABI 3130XL GeneticAnalyser at the SB RAS Genomics Core Facility. The sequence results were analyzed by Chromas Version 2.6.6. The sequences were aligned by BioEdit version 7.2 software. Annotation was performed using Unipro UGENE version 35, BlastN analysis, and the Transposable Elements Platform (TREP) database [24].

### 2.4. Gene Expression Analysis

Total RNA was extracted during two growth stages, GS15 (5 leaves unfolded) and GS31 (1st node) [25], from the leaf apices of the plants using an RNeasy Plant Mini Kit (QIAGEN, Hilden, Germany). RNA samples were then treated with an RNase-free DNase Set (QIAGEN, Germany). cDNA was synthesized using M-MuLV–RH Reverse Transcriptase (Biolabmix, Novosibirsk, Russia). Two previously reported primer sets (Table 1) were used for qRT-PCR analysis: TaFT-F3//R3 and TaFT-B1F//R. Amplification with elongation factor 1-alpha [26] and RNase L inhibitor-like protein [27] primers were used as internal controls. qRT-PCR was carried out using an Applied Biosystems Real-Time PCR System 7500 Fast (Applied Biosystems, Foster City, USA), and the reactions were performed with HS-qPCR Lo-ROX SYBR (2X) (Biolabmix, Novosibirsk, Russia), including three technical replicates per reaction.

## 3. Results

### 3.1. Allelic Composition of Vrn-1, Vrn-3, and Ppd-D1 Alleles in Common Russian Wheat Varieties

Ninety-five common wheat varieties and the ‘Velut’ line were characterized for the allelic composition of vernalization and photoperiod alleles (Table S1). Amplification of genomic DNA using Vrn1AF and Vrn1-INT1R primers (see Table 1) showed that only five varieties harbored the recessive vrn-A1 allele, and one variety had the *Vrn-A1b* allele. The remaining 90 varieties gave an 876 bp and 965 bp PCR product, indicating the presence of the *Vrn-A1a* allele. 

Analysis of the *Vrn-B1* genes showed that among the analyzed genotypes, recessive vrn-B1 was present in 23 varieties, 39 had the *Vrn-B1a* allele, and *Vrn-B1c* was found in 23 varieties. Additionally, six varieties were heterozygous *vrn-B1*/*Vrn-B1a*, two had *Vrn-B1a*/*Vrn-B1c* alleles, and three had *vrn-B1*/*Vrn-B1c*. The dominant *Vrn-D1a* allele was found only in two varieties, with one of them being heterozygous. The rest of the varieties carry the recessive *vrn-D1* allele. Analysis of the *Ppd-D1* genes showed that only one variety had the *Ppd-D1a* (photoperiod-insensitive) allele, and the remaining varieties had *Ppd-D1b*.

Genotyping with primer sets FT-B-INS-F//R indicated that only 2 out of 96 spring wheat varieties had the dominant *Vrn-B3a* allele (Kuibyshevskaya 2, Kazachka) (Table S1). PCR screening with the primer set FT-B-NOINS-F//R indicated that 82 wheat varieties had the recessive *vrn-B3* allele (PCR fragment length 1140 bp), and 10 varieties (Altaiskaya 99, Novosibirskaya 29, Novosibirskaya 67, Novosibirskaya 89, Obskaya 14, Kantegirskaya 89, Aleksandrina, Mana 2, Maria, Omskaya 23) had a novel allele (PCR fragment length 1300 bp). Primer sets FT-B-NOINS-F//R failed to amplify a PCR product in the ‘Velut’ line.

**Figure 1:**
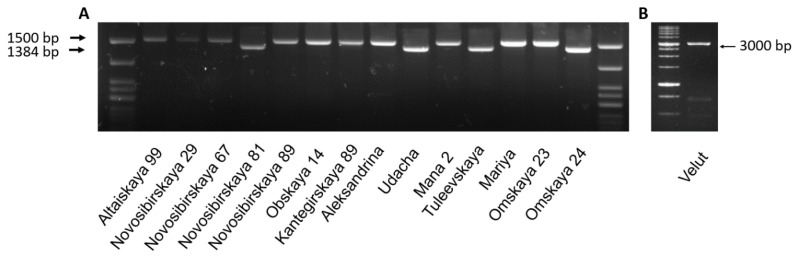
(**A**) PCR amplification using FTpr-F//R primers differentiating *vrn-B3* (1384 bp) and *Vrn-B3e* (1500 bp) alleles; (**B**) PCR amplification with FTpr-F//R and the ‘Velut’ line.

Therefore, the newly specific primer set FTpr-F//R was designed based on sequences of DQ890165.1 (complete sequence of the *Vrn-B3* gene, including the coding sequence and the promoter region of *Triticum aestivum* variety CS (Hope7B)), which covered a greater part of the promoter region and the first intron. Using these primers, a PCR product of approximately 3000 bp was obtained for the ‘Velut’ line and nearly 1500 bp for ten varieties with the *Vrn-B3e* allele (Figure 1). Varieties with the recessive *vrn-B3* allele had a PCR product of approximately 1380 bp.

### 3.2. Sequence Analysis of the Two Novel Vrn-B3 Alleles

**Figure 2:**
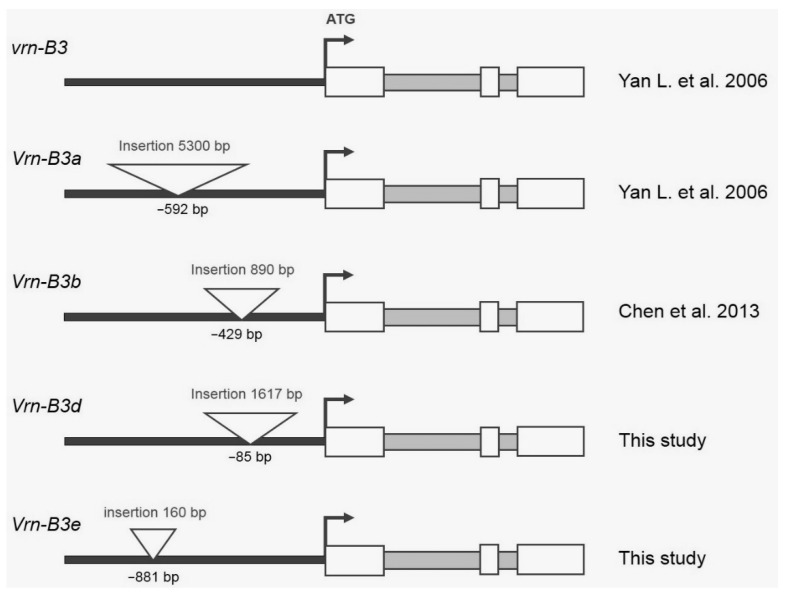
Allelic variations of the *Vrn-B3* gene identified in wheat varieties in this study. The white triangles represent insertions, the white rectangles represent exons, and the black arrows represent the transcriptional start.

The PCR fragment of approximately 3000 bp length obtained in the ‘Velut’ line was sequenced from both directions using an extra set of allele-specific primers described in Supplementary Table S2. Sequence analysis of the *Vrn-B3* variant of ‘Velut’ indicated that a 1617 bp fragment was inserted 85 bp upstream of the start codon (Figure 2). The new *Vrn-3* allele was designated *Vrn-B3d* (submitted to NCBI No: MZ576251). Analysis of the sequence was performed using the UniPro UGENE toolkit. It was found that the fragment inserted in the promotor has identical direct LTRs that are 316 bp in length. The Transposable Elements Platform (TREP) search revealed that the closest homolog was the unannotated *Hordeum vulgare* L. LTR retrotransposon RLX_Hvul_Dacia_RND-1.

The products of approximately 1500 bp obtained from varieties Altaiskaya 99, Novosibirskaya 29, Novosibirskaya 67, Novosibirskaya 89, Obskaya 14, Kantegirskaya 89, Aleksandrina, Mana 2, Maria, and Omskaya 23 were sequenced using the FTpr-F primer. Sequencing confirmed that all of these varieties contained an identical insertion of 160 bp located in the promoter region of the *Vrn-B3* gene 881 bp upstream of the start codon (Figure 2). This allele was designated *Vrn-B3e* (submitted to NCBI No: MZ576252). The insertion sequence shows high homology to a number of regions of the wheat genome (BLASTN analysis relative to the IWGSC 2.0 annotation). These regions are currently not annotated.

### 3.3. Development of Specific Markers for Detecting Novel Vrn-B3 Alleles

**Figure 3:**
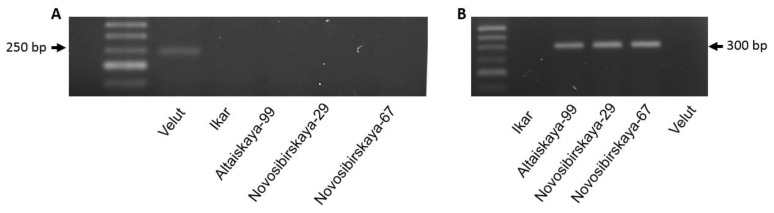
PCR products obtained with the Vrn-B3d-F//R (**A**) and Vrn-B3e-F//R (**B**) markers.

Based on the sequences of *Vrn-B3d* (NCBI No. MZ576251) and *Vrn-B3e* (MZ576252), (Table 1), the *Vrn-B3e* allele was identified by a 302 bp product amplified using the primers Vrn-B3e-F//R, whereas the primer pair Vrn-B3d-F//R with an expected product size of 243 bp was used to detect the *Vrn-B3d* allele (Figure 3). These primer sets for novel alleles can be used to facilitate the detection of *Vrn-B3* alleles and homozygous/heterozygous plants during marker-assisted selection.

### 3.4. The Effects of the Vrn-B3d and Vrn-B3e Alleles on Wheat Heading Time

**Figure 4:**
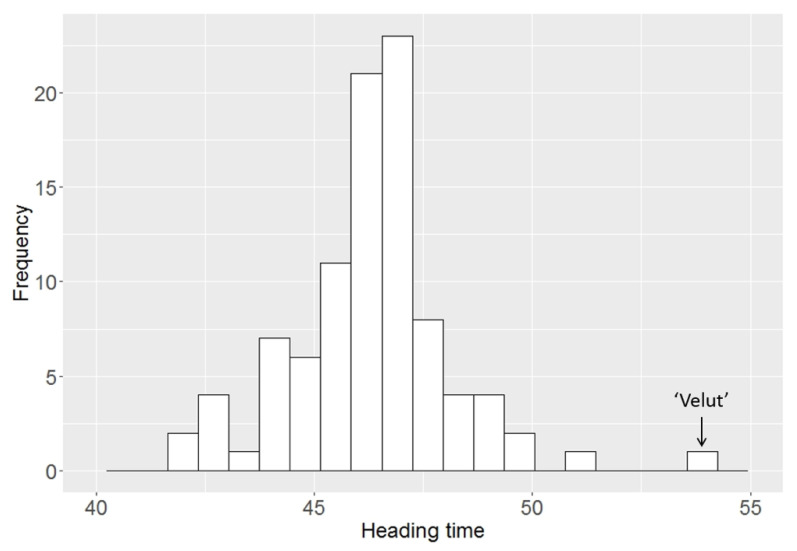
Mean heading times in the studied wheat varieties in 2016 and 2017.

The results of the phenotypic analysis are presented in Figure 4. The mean heading time estimated over two years in the studied population varied from 42.1 to 54.0 days. Varieties with the earliest heading were Provinciya, Polushko, and Tarskaya. The latest-heading varieties were ‘Velut’, Kinelskaya 60, Novosibirskaya 91, and Turinskaya. On average, the plants headed 46.3 days from sprouting.

Phenotypic data were used to measure the differences among days to heading between two groups with certain combinations of vernalization and photoperiod alleles (*Ppd-D1b*/*Vrn-A1a*/*vrn-D1*/*vrn-B3* and *Ppd-D1b*/*Vrn-A1a*/*vrn-D1*/*Vrn-B3e*). Three of ten varieties with *vrn-B3e* had the recessive *vrn-B1* allele, three had *Vrn-B1a,* three had *Vrn-B1c*, and one had a *vrn-B1/Vrn-B1a* allelic combination.

**Figure 5:**
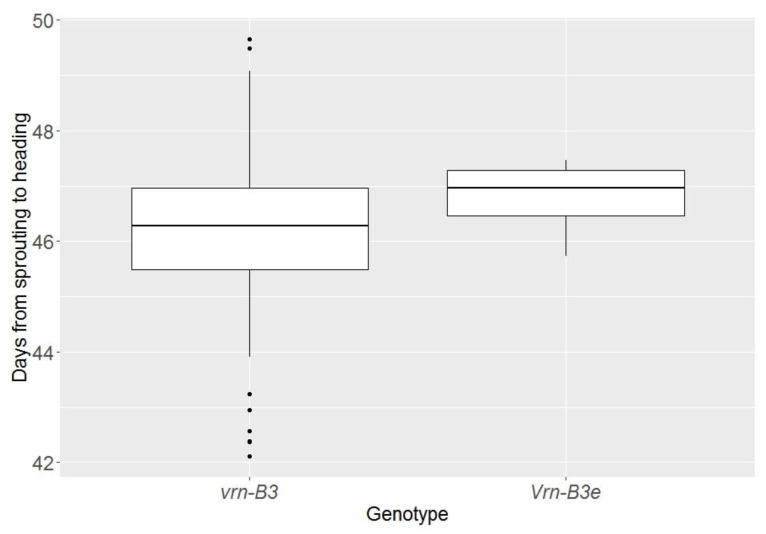
Boxplot of the heading times in groups of plants with *vrn-B3* and *Vrn-B3e* alleles.

We found that varieties with the combination of *Ppd-D1b*/*Vrn-A1a*/*vrn-D1*/*Vrn-B3e* headed, on average, 1.5 days later than varieties with the combination of *Ppd-D1b*/*Vrn-A1a*/*vrn-D1*/*vrn-B3* under long-day conditions (Figure 5). Therefore, it can be assumed that the new *Vrn-B3e* allele delays the heading time even under long-day conditions. However, further investigation is required to assess the effect of a given allele on the heading time.

### 3.5. The Vrn-B3d Allele: Expression-Level Analysis

In the quantitative PCR experiment, we used the F_2_ population from the cross ‘Velut’ (the new *Vrn-B3d* allele) × Tobolskaya (wild-type *vrn-B3*). These varieties were tested for allelic variations at the *Vrn-A1*, *Vrn-B1*, *Vrn-D1*, and *Ppd-D1* loci (the major determinants of flowering time). The ‘Velut’ and Tobolskaya varieties have the same *Ppd-D1b*, *Vrn-B1c*, and *vrn-D1* alleles, but they differ in their *Vrn-A1* alleles (*Vrn-A1a* and *Vrn-A1b*, respectively) (Table S1).

We analyzed F_2_ hybrids using sequence-specific markers to select homozygous plants carrying the *Vrn-B3d* or *vrn-B3* allele (we used Vrn-B3d-F//R (this study) and FT-B-NOINS [2] primers for screening). Ten biological replicates were sampled, and each plant represented an individual replicate. All selected plants were heterozygous for the *Vrn-A1* loci (initially, we planned to use homozygous plants with the dominant *Vrn-A1* allele, but there were not enough such plants in our sample). Expression was investigated in leaf tissue sampled at two growth stages (five leaves unfolded and the first node) under short-day conditions (9 h of light). The genes we used as an internal control showed specific and efficient amplification, while the expression level of the target gene turned out to be both weak and nonspecific. This implies that there is no difference in the expression level or heading time between plants with *vrn-B3* and *Vrn-B3d* alleles.

## 4. Discussion

Allelic diversity within genes controlling flowering time is one of the most important factors contributing to the adaptability and productivity of wheat. In the present study, allelic variation at these loci was tested for 95 spring wheat varieties grown in Siberia.

The genetic diversity within the studied genotypes is mainly based on the *Vrn-B1* and *Vrn-B3* loci. The dominant alleles of the *Vrn-B1* gene (*Vrn-B1a* or *Vrn-B1c*) predominated. In the current study, the dominant *Vrn-B1* was found in 76% of varieties. Low variation was observed at the *Vrn-A1*, *Vrn-D1*, and *Ppd-D1* loci. This agrees with a previously conducted study [28] of 48 Siberian spring common wheat varieties, where varieties with the dominant *Vrn-B1* haplotypes were the most frequent. Shcherban et al. (2015) reported screening 245 predominantly spring wheat varieties for the photoperiod and vernalization response genes in common wheat varieties from different regions of Europe [29]. As expected, insensitivity to the photoperiod *Ppd-D1a* allele was mostly found in wheat from southern regions (32%), while in central, western, and northern regions, the *Ppd-D1a* allele was more frequent (98.8% and 95.6%, respectively). In the same study, *Vrn-A1a* together with the dominant alleles of *Vrn-B1* (mostly *Vrn-B1a*) and recessive *vrn-D1* were found in nearly half of the varieties. The *Vrn-A1a* allele, either alone or with the dominant *Vrn-B1*, also predominated in wheat genotypes from Canada and the USA [30,31]. The strong presence of the dominant *Vrn-A1* and/or *Vrn-B1* in high latitude spring wheat varieties has been reported multiple times. Under these growing conditions, this genetic background causes an earlier heading time, increasing the adaptability of plants in areas with a short growing season.

The presence of TEs can influence plant gene expression through several potential mechanisms. TE insertions into the promoter or nearby regulatory regions may disrupt the existing regulation of genes, resulting in changes in the expression patterns. Several studies have found such dominant alleles of the *Vrn-B3* gene in wheat.

Dominant *Vrn-B3* alleles are not common in wheat germplasm, although the *Vrn-B3a* allele was detected widely in the cultivated varieties of *T. durum* from Ukraine and Russia [32]. This allele was first identified in the common wheat variety Hope, which was derived from a cross between the emmer wheat variety ‘Yaroslav’, the donor of *Vrn-B3a* in Hope, and the Canadian *T. aestivum* variety ‘Marquis’ [2,33,34]. Subsequently, this allele was found in three varieties of *T. aestivum* from China [16,35]. Although the *Vrn-B3a* allele is rare in common wheat, the analysis of 214 varieties from different ecogeographical areas showed that the dominant allele was abundant among tetraploid wheat *T. durum* and *T. dicoccum* originating from Ukraine and Russia. It was demonstrated that the *Vrn-B3a* allele is associated with early flowering under both long-day and short-day conditions [2]. The *Vrn-B3c* allele has two small deletions (4 bp and 20 bp) inside of a retrotransposon detected in Hope [16]. The *Vrn-B3b* allele is characterized by an insertion of 890 bp within the promoter, resulting in delayed heading. It was assumed that the *Vrn-B3b* allele has a more recent origin (compared to the *Vrn-B3a* allele) in the *T. dicoccum* lineage [34]. Additionally, two novel 1300 bp and 2000 bp fragments were observed from amplification using primers FT-B-NOINS-F and FT-B-NOINS-R (an amplification product of 1140 bp indicates the recessive *vrn-B3* allele) in Iranian wheat landraces [36]. Molecular characterization of these alleles was not performed.

In the present study, marker analysis revealed that only two varieties carried the *Vrn-B3a* allele, and the great majority (86%) of varieties carried the recessive *vrn-B3* allele. During the investigation, two novel *Vrn-B3* allelic variants carrying mutations within the promoter region were found. These varieties were characterized by 160 bp and 1617 bp insertions and were designated *Vrn-B3e* and *Vrn-B3d*, respectively.

The primary structure analysis showed that the *Vrn-B3d* allele has an insertion of a retroelement flanked by identical 316 bp LTRs. The fact that the LTRs are completely identical implies that the insertion occurred recently. Several studies utilizing an approach of comparison of long terminal repeat (LTR) divergences in plant genomes, including hexaploid wheat, showed that the proliferation of retroelements specific to certain genomes occurred in the diploid progenitors of hexaploid wheat [37,38,39]. Thus, the proliferation of the TEs in the genomes of tetraploid and hexaploid wheat is suppressed. According to the scheme of origin of the ‘Velut’ line (Figure S1), insertion of the transposon could have occurred during pollination of the Rodina variety with gamma-irradiated pollen *Ae. speltoides*.

Interestingly, the similarity search with BLASTN against available TE databases found a strong hit to a *Hordeum vulgare* L. LTR retrotransposon (RLX_Hvul_Dacia_RND-1). The retrotransposon was not classified into any known class because no structural motifs were found. These transposons have high DNA sequence similarity in their coding internal domain (identity > 80%) but not in the terminal repeat region. Both of them are presumably nonautonomous because no structural motifs were found in their internal domains. Unlike the previously identified allelic variations of the *Vrn-B3* gene, the one found in the ‘Velut’ line did not have any significant effect on flowering time. Presumably, the insertion region does not include any important regulatory sequences within the promoter.

Ten wheat varieties possessed the novel *Vrn-B3e* allele, which had a smaller insertion within the promoter. Our field data suggest that this allele might be associated with delayed heading. Pedigree analyses performed using the database GRIS (http://www.wheatpedigree.net, accessed on 16 November 2021) for the ten varieties from this study with *Vrn-B3e* indicated that seven of them (Novosibirskaya 67, Novosibirskaya 29, Novosibirskaya 89, Kantegirskaya 89, Obskaya 14, Omskaya 23, Aleksandrina) were derived from a cross of Lutescens 55-11 and Albidum 24 originating in the Volga region (Supplementary Figure S2). Based on the length of the PCR fragment, we can assume that this allele was also found among Iranian wheat landraces [40]. Among 395 wheat landraces studied, Derakhshan et al. (2013) found this allele in 9, both winter and spring genotypes. We suggest that this allele must be widespread in common wheat from different cultivation centers. Overall, our results demonstrate that Russian wheat germplasm could be used as a potential source of genetic diversity for controlling heading times in wheat.

## 5. Conclusions

In this study, we examined allelic composition at the *Ppd-D1*, *Vrn-A1*, *Vrn-B1*, *Vrn-D1*, and *Vrn-B3* loci among Siberian common wheat. Two novel alleles of the *Vrn-B3* gene were found. We showed that *Vrn-B3d* and *Vrn-B3e* alleles carrying 1617 bp and 160 bp insertions, respectively, within the promoter region, were associated with a delay of heading under long-day conditions.

## Figures and Tables

**Table 1:** Primer sequences, annealing temperatures, and expected PCR product sizes.

Primers	Sequence	Gene Region	Annealing Temp. °C	Allelic Variant	Product Size (bp)	Source
Vrn1AF Vrn1-Int1R	gaaaggaaaaattctgctcg gcaggaaatcgaaatcgaag	*VRN-A1* promoter	60	*vrn-A1* *Vrn-A1a* *Vrn-A1b*	734 876 and 965 714	[8]
Ex1/C/F Intr1/B/R3	gttctccaccgagtcatggt ctcatgccaaaaattgaagatga	*VRN-B1* intron-1	58	*Vrn-B1a* *Vrn-B1b* *Vrn-B1c*	1091 1055 705	[9]
Ex1/C/F Intr1/B/R3	gttctccaccgagtcatggt caaatgaaaaggaatgagagca	*VRN-B1* intron-1	60	*vrn-B1*	1531	[9]
Intr1/D/F Intr1/D/R3 Intr1/D/R4	gttgtctgcctcatcaaatcc ggtcactggtggtctgtgc aaatgaaaaggaacgagagcg	*VRN-D1* intron-1	61	*vrn-D1* *Vrn-D1a*	997 1671	[9]
Ppd-D1_F Ppd-D1_R1 Ppd-D1_R2	acgcctcccactacactg gttggttcaaacagagagc cactggtggtagctgagatt	*PPD-D1* promoter	54	*Ppd-D1a* *Ppd-D1b*	288 414	[22]
FT-B-INS-F FT-B-INS-R	cataatgccaagccggtgagtac atgtctgccaattagctagc	*VRN-B3* promoter	57	*Vrn-B3a*	1200	[2]
FT-B-NOINS-F FT-B-NOINS-R	atgctttcgcttgccatcc ctatccctaccggccattag	*VRN-B3* promoter	57	*vrn-B3*	1140	[2]
TaFT-F3 TaFT-R3	cagcagcccagggttgag atctgggtctaccatcacgagtg	*VRN-B3* exons 1 and 2	60	*-*	72	[2]
TaFT-B1-F TaFT-B1-R	gtcgttcgggcaggag tggaagagtacgagcacga	*VRN-B3* exon 3	60	*-*	81	[23]
FTpr-F FTpr-R	cgaaagcggagggtatattaaa cccgaacatagaagaagcatag	*VRN-B3* promoter	60	*vrn-B3* *Vrn-B3e* *Vrn-B3d*	1384 1544 3001	This study
Vrn-B3e-F Vrn-B3e-R	cgccagacagcattcctatta tactggtgtctcttgccattc	*VRN-B3* promoter	59	*Vrn-B3e*	302	This study
Vrn-B3d-F Vrn-B3d-R	gttacaccacatccacagaac ccaatactaccggccactaa	*VRN-B3* promoter	62	*Vrn-B3d*	243	This study

## Data Availability

The data presented in this study are available in Supplementary Materials. The sequencing data have been submitted to the NCBI database (accession numbers MZ576251 and MZ576252).

## Supplementary Materials

The following are available online at https://www.mdpi.com/article/10.3390/biom11121897/s1, Table S1: Heading date and allelic composition of the *Vrn-1*, *Vrn-3*, and *Ppd-1* genes in the studied Russian common wheat varieties. Phenotyping was performed in 2016–2017, Table S2: Primer sets used for sequencing of the *Vrn-B3d* allele promoter, Figure S1: The origin of the ‘Velut’ line with a delayed heading time, Figure S2: The phylogenetic relationships and origins of the varieties carrying the novel *Vrn-B3e* allele (the varieties are given in bold).
