# Supplementary material for: Allelic Variation Analysis at the Vernalization Response and Photoperiod Genes in Russian Wheat Varieties Identified Two Novel Alleles of Vrn-B3
Source: Biomolecules. 2021 Dec 17;11(12):1897. doi: 10.3390/biom11121897 (PMC8699075; doi:10.3390/biom11121897)
Supplement: Supplementary file 1 [file biomolecules-11-01897-s001.zip › Figure S1.pptx]

## Slide 1
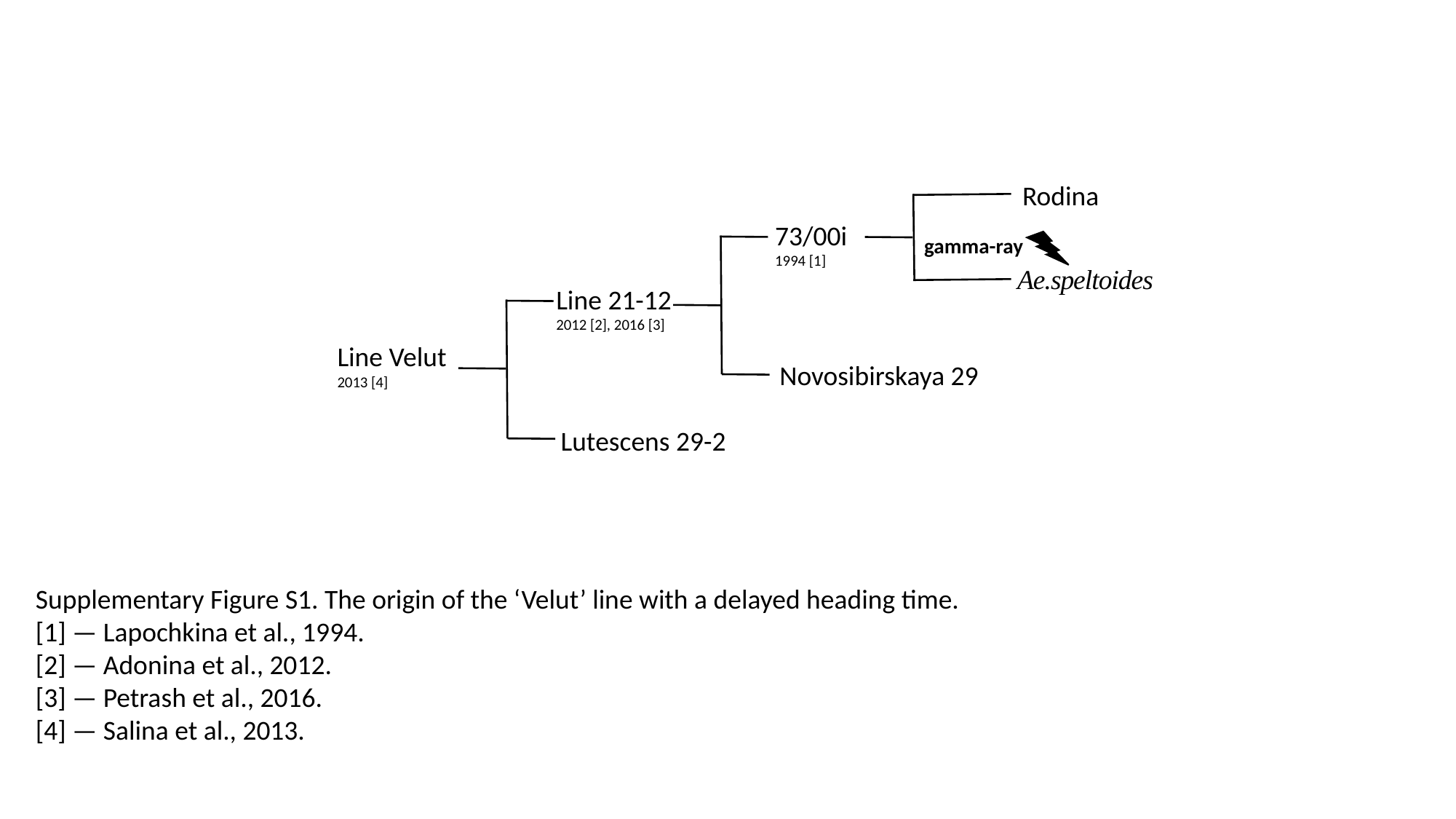

Rodina
73/00i
1994 [1]
gamma-ray
 Ae.speltoides
Line 21-12
2012 [2], 2016 [3]
Line Velut
2013 [4]
Novosibirskaya 29
Lutescens 29-2
Supplementary Figure S1. The origin of the ‘Velut’ line with a delayed heading time.
[1] — Lapochkina et al., 1994.
[2] — Adonina et al., 2012.
[3] — Petrash et al., 2016.
[4] — Salina et al., 2013.
