# Supplementary material for: Allelic Variation Analysis at the Vernalization Response and Photoperiod Genes in Russian Wheat Varieties Identified Two Novel Alleles of Vrn-B3
Source: Biomolecules. 2021 Dec 17;11(12):1897. doi: 10.3390/biom11121897 (PMC8699075; doi:10.3390/biom11121897)
Supplement: Supplementary file 1 [file biomolecules-11-01897-s001.zip › Figure S2.pptx]

## Slide 1
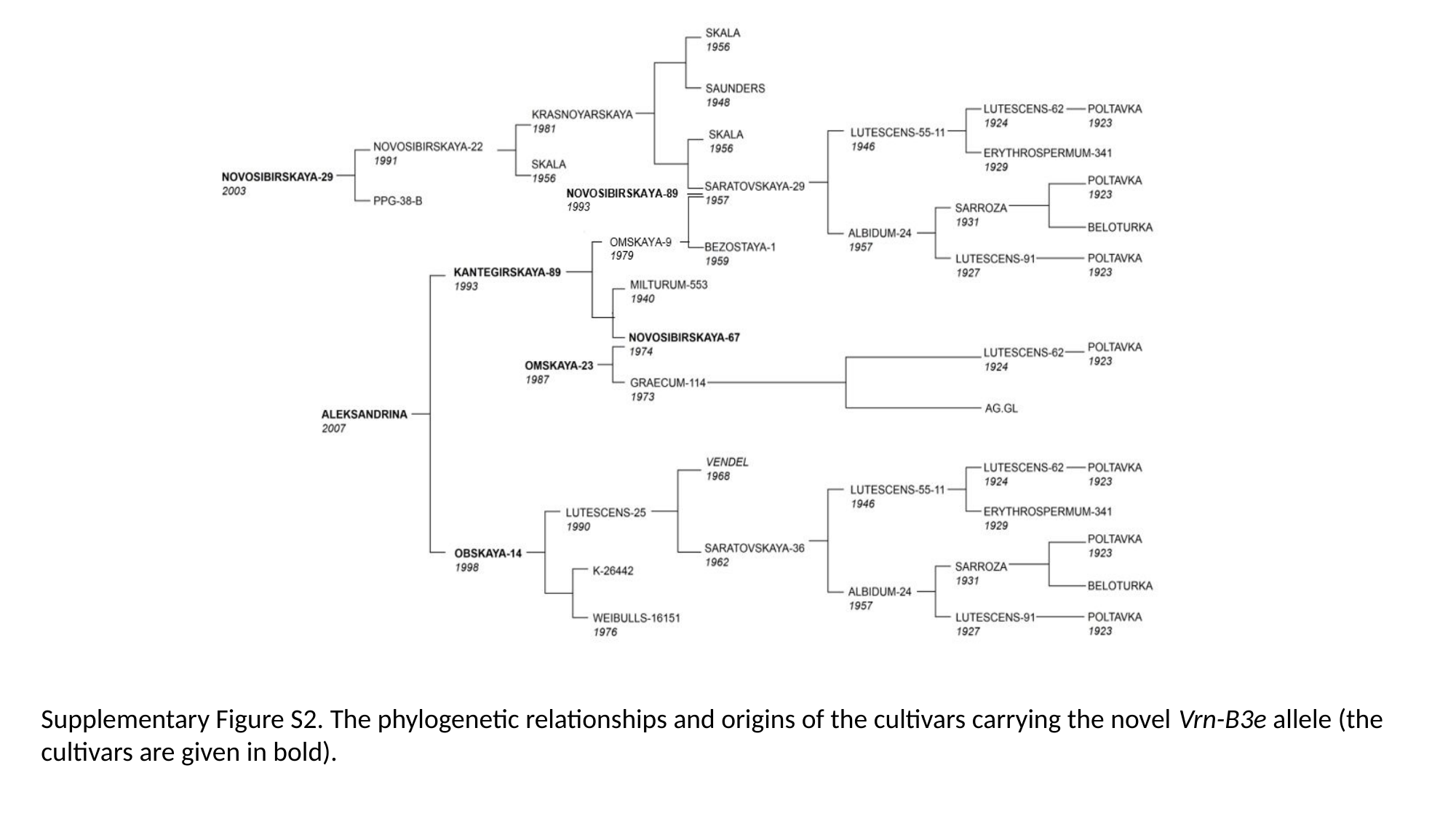

Supplementary Figure S2. The phylogenetic relationships and origins of the cultivars carrying the novel Vrn-B3e allele (the cultivars are given in bold).
